# Why Don’t More Mitochondrial Diseases Exhibit Cardiomyopathy?

**DOI:** 10.3390/jcdd10040154

**Published:** 2023-04-01

**Authors:** Nina Singh, Mindong Ren, Colin K. L. Phoon

**Affiliations:** 1 Division of Pediatric Cardiology, Department of Pediatrics, New York University Grossman School of Medicine, New York, NY 10016, USA; nina.singh@nyulangone.org; 2 Department of Anesthesiology, New York University Grossman School of Medicine, New York, NY 10016, USA; mindong.ren@nyulangone.org; 3 Department of Cell Biology, New York University Grossman School of Medicine, New York, NY 10016, USA

**Keywords:** cardiomyopathy, mitochondrial disease, mouse models, oxidative phosphorylation

## Abstract

Background: Although the heart requires abundant energy, only 20–40% of children with mitochondrial diseases have cardiomyopathies. Methods: We looked for differences in genes underlying mitochondrial diseases that do versus do not cause cardiomyopathy using the comprehensive Mitochondrial Disease Genes Compendium. Mining additional online resources, we further investigated possible energy deficits caused by non-oxidative phosphorylation (OXPHOS) genes associated with cardiomyopathy, probed the number of amino acids and protein interactors as surrogates for OXPHOS protein cardiac “importance”, and identified mouse models for mitochondrial genes. Results: A total of 107/241 (44%) mitochondrial genes was associated with cardiomyopathy; the highest proportion were OXPHOS genes (46%). OXPHOS (*p* = 0.001) and fatty acid oxidation (*p* = 0.009) defects were significantly associated with cardiomyopathy. Notably, 39/58 (67%) non-OXPHOS genes associated with cardiomyopathy were linked to defects in aerobic respiration. Larger OXPHOS proteins were associated with cardiomyopathy (*p* < 0.05). Mouse models exhibiting cardiomyopathy were found for 52/241 mitochondrial genes, shedding additional insights into biological mechanisms. Conclusions: While energy generation is strongly associated with cardiomyopathy in mitochondrial diseases, many energy generation defects are not linked to cardiomyopathy. The inconsistent link between mitochondrial disease and cardiomyopathy is likely to be multifactorial and includes tissue-specific expression, incomplete clinical data, and genetic background differences.

## 1. Introduction

Traditionally, we think of mitochondria as the “powerhouses” of the cell. It is widely stated that patients with mitochondrial diseases have cardiac manifestations because the heart is a high energy-requiring organ [1,2,3,4,5]. However, only 20–40% of children with mitochondrial diseases exhibit cardiomyopathies [1,6,7]. This is partially due to incomplete penetrance [8] of cardiomyopathy in mitochondrial diseases, but incomplete penetrance alone does not explain the whole picture. Indeed, many mitochondrial diseases, such as Acyl-CoA dehydrogenase, medium-chain deficiency (ACADMD), X-linked recessive Charcot–Marie–Tooth disease-4, and combined oxidative phosphorylation deficiency 27, do not exhibit cardiomyopathy at all [9]. The absence of any cardiac manifestations in patients with these mitochondrial diseases implies that a mechanistic approach may reveal new insights into what causes cardiomyopathy in some mitochondrial diseases but not others.

In addition to energy production, mitochondria play many additional, critical roles in cellular physiology, including cell signaling, trafficking, stress responses, and apoptosis, and interact with many other intracellular organelles such as the endoplasmic reticulum, lysosomes, and peroxisomes [10,11,12,13,14,15,16]. Myocardial maturation requires normal mitochondrial function; evidence points to reactive oxygen species (ROS) as being important in signaling [17,18,19,20,21,22]. Their varied roles in critical cell processes, including in myocardial development, make it all the more surprising why only a minority of mitochondrial diseases seem to exhibit cardiomyopathy.

In the rapidly evolving field of mitochondrial diseases, Falk and colleagues’ recently released, comprehensive Mitochondrial Diseases Gene Compendium [9] provides a unique opportunity to ask the question: What distinguishes mitochondrial diseases that present with cardiomyopathy from those that do not? In this paper, we systematically looked for functional differences in genes underlying mitochondrial diseases that do versus do not cause cardiomyopathy. We supplemented our analysis of the Mitochondrial Diseases Gene Compendium with published mouse model data. Our approach was to look at proportions of genes, not patients, to define those gene functions that are most important to normal cardiac function.

## 2. Materials and Methods

### 2.1. Data Collection

The original list of 256 genes and their descriptions was sourced from Falk and colleagues’ Mitochondrial Disease Genes Compendium [9]. This Compendium was developed and curated by a team of experts in mitochondrial disease and includes a wealth of data for each gene, including both gene function and localization, as well as clinical disease specifics. The data are based on an informatics analysis of genetic and disease information available in the Mitochondrial Disease Sequence Data Resource (MSeqDR)-LSDB and ClinVar as of February 2019 [9]. We note that this Compendium does not include either the number of patients or the specific variants for each gene.

We did not attempt to associate specific gene variants (an enormous number, coupled with the number of genes) with cardiomyopathy; rather, we examined data at the gene level to find possible associations with the binary categorization (as determined in the Compendium itself) of cardiomyopathy versus no cardiomyopathy. To search for differences between mitochondrial diseases with and without cardiomyopathy, we examined multiple Compendium data fields for each gene. These fields included “Gene Name”, “Protein or RNA Localization”, “Protein Function”, “Genome Origin”, and “Organ System Manifestations” (Table S1). If the Protein or RNA localization field of the Compendium was “Other” or not clearly mitochondrial, we examined the associated gene more closely to determine whether it was associated with primary or secondary mitochondrial dysfunction. Based on additional curated informatics data from MitoMiner 4.0 (a database of mitochondrial genes with information from public resources) [23,24], genes were eliminated if they were known or predicted to be non-mitochondrial. After this process, 241 of the original 256 genes were retained.

The protein function field of the Compendium was examined and categorized into “Oxidative Phosphorylation (OXPHOS)”, “mtDNA Gene Expression”, “Metabolism”, “Solute Carrier Family”, “Protein Targeting to Mitochondria”, and “Other” categories, as described by Mootha and colleagues [25]. The presence or absence of central nervous system manifestations and cardiac manifestations in each disease was noted from the Compendium’s “Organ System Manifestations” field. In the select cases where these manifestations were unclear based on the description from the Compendium, OMIM [26] was consulted to classify the manifestations.

Cardiac manifestations were further subclassified into “Cardiomyopathy” and “Other”. Each cardiac manifestation that was classified as “Other” was further examined to confirm that it was not secondary to a cardiomyopathy, through a combination of reading the Compendium’s references and our own PubMed search. If the papers did not show any evidence of primary cardiac manifestations, the entry corresponding to that gene in our dataset was changed to indicate no cardiac manifestations.

Based on our findings from the initial analysis, we conducted a further subanalysis of the non-OXPHOS proteins that caused cardiomyopathy to understand whether the mechanism of cardiomyopathy for these genes was also likely to be deficient in energy generation (Table S2). We searched PubMed using the search terms “(GENE) AND ((OXPHOS) OR (ATP generation))” and noted whether any search results linked these non-OXPHOS genes to aerobic respiration deficits. We also conducted a further subanalysis of the OXPHOS proteins. We used SWISS-MODEL [27,28] (https://swissmodel.expasy.org/repository/uniprot/; accessed on 2 December 2020) to identify the number of amino acids for each protein encoded and BioGRID [29,30] (https://thebiogrid.org/; accessed on 20 February 2023) to identify the number of protein interactors. These were investigated as surrogates of protein “importance”.

Mouse models could provide additional insight into the importance of a specific mitochondrial protein in cardiac function. We searched PubMed using the search terms “[gene of interest] + mouse” and/or “[gene of interest] + mouse + heart” (https://pubmed.ncbi.nlm.nih.gov; accessed on 28 February 2023) for each gene in the Compendium, then culled to show only those with mouse models. We further culled to show only those mouse models in which a cardiac phenotype was determined (clear cardiac phenotype) or possible (only biochemical/cellular data but not a clear cardiac phenotype); ”not determined” models were omitted from the prior dataset (Table S3). To broaden our search further for mouse models of mitochondrial disorders, we mined the International Mouse Phenotyping Consortium (IMPC) [31,32] (https://www.mousephenotype.org/; accessed on 27 February 2023) and looked for additional evidence of cardiac phenotypes, along with premature lethality (pre-weaning and embryonic lethality) among mitochondrial genes.

### 2.2. Analysis

Fisher’s exact test was used to compare the following parameters between mitochondrial disease-causing genes with versus without cardiomyopathy: localization category, gene classification, genome origin, nuclear or mitochondrial DNA, and central nervous system manifestations. We calculated the odds ratios and confidence intervals for having cardiomyopathy versus not by gene category, which was the only parameter that Fisher’s exact test found to be significantly different between mitochondrial disease-causing genes with versus without cardiomyopathy. Gene categories with more than five mitochondrial diseases with and without cardiomyopathy were further subdivided and analyzed. For example, this led to a subanalysis of diseases in the OXPHOS category (I, II, III, IV, V, accessory), mtDNA gene expression category (tRNA, tRNA synthetase, ribosome component, nucleotide modification, other), and metabolism category (amino acid metabolism, mitochondrial fatty acid metabolism, Krebs/TCA cycle, phospholipid metabolism, nucleotide metabolism, intermediary metabolism, other). Any *p*-values < 0.05 were considered to be statistically significant.

In our subanalysis of OXPHOS proteins, we used Student’s *t*-test to compare the average number of amino acids (surrogate for “importance”) and the average number of interactors (surrogate for “importance”) across genes with versus without cardiomyopathy. We performed a linear regression analysis between the number of amino acids and the number of interactors. We examined mouse models for concordance between the presence or absence of cardiomyopathy in mouse models versus human patients.

## 3. Results

Of the 241 mitochondrial disease-causing genes examined, 83 (34%) were due to oxidative phosphorylation mutations, 74 (31%) were due to mtDNA gene expression mutations, 52 (22%) were due to metabolism mutations, 16 (7%) were due to solute carrier family mutations, 4 (2%) were due to protein targeting to mitochondria mutations, and 12 (5%) were due to other mutations (Table 1). In total, 204 (85%) mitochondrial disease-causing genes were caused by mutations in nuclear DNA and 37 (15%) were caused by mutations in mitochondrial DNA. Cardiac manifestations were seen in 119 (49%) of the mitochondrial disease-causing genes examined and central nervous system manifestations were seen in 224 (93%) of the mitochondrial disease-causing genes examined. Of the cardiac manifestations seen, 107 (90%) were cardiomyopathies and 12 (10%) were other cardiac manifestations (e.g., pulmonary hypertension, second-degree heart block).

**Figure 1:**
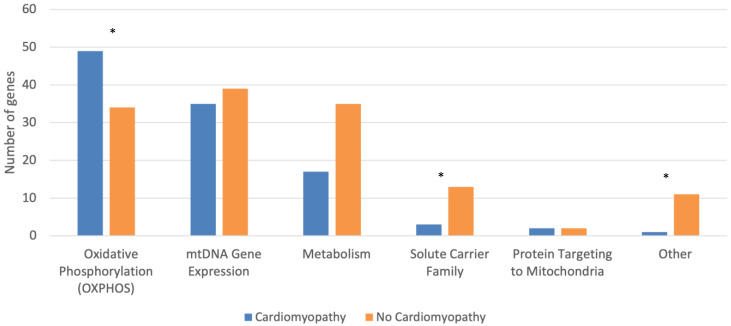
Mitochondrial Disease-Causing Genes Classified by the Presence of Cardiomyopathy and Protein Function Affected, as Defined by Mootha and Colleagues [25]. * indicates *p* < 0.05.

We performed a statistical analysis to compare the characteristics of mitochondrial disease-causing genes with and without cardiomyopathy, but it should be noted that the numbers are very low and statistical results should be interpreted with caution. We found no differences in the localization, genome origin (origin by chromosome), nuclear gene, or central nervous system parameters between mitochondrial disease-causing genes with and without cardiomyopathy. However, there was a significant difference between the types of genes underlying mitochondrial diseases with and without cardiomyopathy (*p* < 0.001) (Table 1 and Figure 1). Specifically, mutations in the oxidative phosphorylation category were more prevalent among genes with cardiomyopathy than those without cardiomyopathy (*p* = 0.001, OR = 2.48, CI: 1.44–4.28). Mutations in the solute carrier family category and other category were less prevalent among genes with cardiomyopathy than those without cardiomyopathy (*p* = 0.038, OR = 0.27, CI: 0.07–0.97 and *p* = 0.014, OR = 0.11, CI: 0.01–0.83), respectively. There were no significant differences in the prevalence of mutations in the mtDNA gene expression, metabolism, and protein targeting to mitochondria categories between genes with and without cardiomyopathy. Taken together, these results support an association between deficits in energy production and cardiomyopathy.

Upon further subanalysis of gene categories with more than five mitochondrial diseases with and without cardiomyopathy, there was a difference in the prevalence of different OXPHOS complex mutations in genes with underlying OXPHOS defects (*p* < 0.020, Table 1). Notably, 0/5 genes with Complex III defects and 4/4 genes with Complex V defects resulted in cardiomyopathy, suggesting that Complex III may be less important in cardiac function and Complex V may be very important. There were no differences seen in the prevalence of different subcategories of mtDNA gene expression mutations (Table 1) or metabolism mutations (Table 1), with the exception of a difference in mitochondrial fatty acid metabolism mutations. Of the diseases with these mutations, seven presented with cardiomyopathy and three did not (*p* = 0.009). Our subanalysis of non-OXPHOS genes that cause cardiomyopathy revealed that 39/58 genes (67%) were associated with known defects in aerobic respiration (Table S2); in other words, although these genes are not categorized as “OXPHOS” genes, they ultimately affect OXPHOS. These results further support an association between deficits in energy production and cardiomyopathy. However, it is notable that a substantial proportion of genes associated with energy production were not linked with cardiomyopathy (e.g., 34/83 (41%) of OXPHOS genes).

We further analyzed OXPHOS proteins to understand what differentiated those that caused cardiomyopathy from those that did not. We used number of amino acids and number of physical interactors as surrogates for importance to see if there was a link between gene “importance” and a likelihood of causing cardiomyopathy. Our subanalysis of OXPHOS proteins found that larger OXPHOS proteins were weakly associated with cardiomyopathy (*p* < 0.05), although the number of protein interactors was not (Figure S1a,b). We found that the number of amino acids correlates weakly (but is statistically significant) with the number of physical interactors (proteins, genes) (r = 0.38, *p* < 0.0001) (Figure S1c). The association between larger OXPHOS proteins and cardiomyopathy was likely due to larger genes having more possible locations to have mutations and thus present with clinical cases, including those that demonstrate cardiomyopathy. The weak correlation between the number of amino acids and number of physical interactors suggests that these measures may not be the best surrogates for protein “importance”.

To gain further insights into both the potential for human cardiomyopathy and mechanisms underlying cardiomyopathic phenotypes, we performed a systematic search for mouse models of mitochondrial disorders associated with the genes in the Compendium. Animal studies provided more data, especially in diseases with few reported human patients, and often provided more mechanistic detail since the methods were more focused on rigorous multi-level biological analyses. We searched specifically for mouse models that exhibited cardiomyopathic abnormalities, either on a cellular level (e.g., biochemical derangements, abnormal mitochondrial functioning) or tissue/organ level (e.g., electron microscopy, histology, echocardiography). Cardiomyopathy present in either human patients or mouse models was labeled as “TRUE” while the absence of cardiomyopathy despite testing in mouse models was labeled as “FALSE”. There were 23 “TRUE [human]-TRUE [mouse]” pairs, which may offer mechanistic insights into the human cardiomyopathies (Table S3). We also found 26 “FALSE [human]-TRUE [mouse]” pairs (Table S3), which likely reflect that most mouse models are knockout models and therefore, more severely affected. There were very few “TRUE-FALSE” pairs, which likely reflects the lack of assays performed on the mouse model (the scientific focus of the research lab studying the mouse).

These mouse data indicate we are likely underestimating the number of human gene mutations associated with cardiomyopathy and should complete cardiac screens of human patients with mitochondrial diseases more routinely. An examination of the mouse data showed that the pathophysiological mechanisms include abnormal respiratory complex formation and activity, reduced ATP production, mitochondrial ultrastructural abnormalities (including in the cristae), and abnormalities of sarcomere function and calcium regulation. Three out of the 34 OXPHOS genes that did not demonstrate cardiomyopathy in humans did demonstrate cardiomyopathy in mice (*Ndufa13*, *Bcs1l*, *Aifm1*). Twelve of the 52 mouse models identified with cardiomyopathy (*Acadv1*, *Fxn*, *Mto1*, *Taco1*, *Hmgcs2*, *Mpv17*, *Opa1*, *Pnpla8*, *Tmem126b*, *Gpd2*, *Mpv17*, *Micu1*) showed that the presence of cardiomyopathy can be dependent on the degree of stress. These findings could present valuable opportunities to study human cardiac function in more detail and to perform more careful observation of humans with these cardiac diseases.

Finally, data mining of the IMPC database (https://www.mousephenotype.org; accessed on 27 February 2023) revealed 47 additional mouse models not found in our systematic PubMed search, but many more genes (103/118) were not associated with a significant cardiac phenotype. The paucity of a cardiomyopathic phenotype in many of these mouse knockout models almost certainly represents the nature of the screening phenotyping pipeline of the IMPC, which would not include important biochemical data. Notably, many gene knockouts were associated with early (pre-weaning or embryonic) lethality, which may suggest possible cardiac lethality very early in life (Table S4).

## 4. Discussion

Our investigation was initiated by the enigmatic finding that only 20–40% of children with mitochondrial diseases have cardiomyopathy [1,6,7]. Given that the mechanism of cardiomyopathy in mitochondrial diseases is thought to be due to defects in energy generation, and that the heart is a high-energy-requiring organ, it is unclear why more patients with mitochondrial disease do not have cardiomyopathy.

In our analysis, only 107/241 (44%) of all affected genes known to cause mitochondrial disease were associated with cardiomyopathy. Energy (ATP) generation appeared to be most importantly associated with cardiomyopathy, as reflected in the OXPHOS and fatty acid metabolism gene defects representing the highest proportion of genes causing cardiomyopathy (52%) and these gene categories having a significant association with cardiomyopathy. Furthermore, our PubMed search of non-OXPHOS genes that caused cardiomyopathy revealed that 39/58 (67%) were linked to deficits in energy production. Nevertheless, a large number of defects associated with ATP generation were not associated with cardiomyopathy, such as the 34/83 (41%) of OXPHOS genes that were not associated with cardiomyopathy.

The prior literature has focused on the causative mutations underlying all mitochondrial diseases [5,9] or the genes underlying mitochondrial diseases with cardiac manifestations [33] rather than asking the question of what differentiates mitochondrial diseases that cause cardiomyopathy from those that do not. An analysis of 289 mitochondrial disease-causing genes found that 64% had a primary role specific to OXPHOS biogenesis [5]. It further noted that as disease gene discovery has used more massively parallel sequencing, the number of genes with indirect roles in OXPHOS has increased to about half of all new results [5]. In a recent analysis of mitochondrial diseases causing cardiac manifestations, 34.3% of genes were found to encode for key proteins involved in OXPHOS [33].

These findings, combined with our new results, are only partially consistent with the widely believed hypothesis that cardiomyopathy in mitochondrial diseases results from deficits in energy generation [1,2,3,4,5]. Mitochondrial defects often cause perturbations in aerobic respiration, and insufficient energy production is thought to increase oxidative stress on the heart (a high-energy requiring organ) and leads to downstream effects such as mitochondrial proliferation, reactive oxygen species damage, and hypertrophy [3,34]. Cardiomyopathy, and specifically hypertrophic cardiomyopathy (HCM), is the most common cardiac manifestation seen in mitochondrial disease [6,15,34]. Scaglia and colleagues found that of 45 patients with cardiac manifestations of mitochondrial disease, 58% had hypertrophic cardiomyopathy [6]. Mastantuono and colleagues later found HCM to be associated with ~90 mitochondrial disease genes out of 100 with cardiac manifestations [15]. Other cardiac manifestations of mitochondrial disease including other cardiomyopathies and abnormalities in the cardiac conduction system, valves, coronary arteries, and connective tissue can also occur [9,15,34,35].

This diversity of possible cardiac manifestations other than cardiomyopathy may partially reflect the diversity of roles that mitochondria play. In addition to energy production, mitochondria are involved in signaling, regulation of reactive oxygen species, trafficking, apoptosis, iron metabolism, and calcium homeostasis [10,11,12,13,14,15]. These other possible roles could lead to other mechanisms of cardiomyopathy development, such as defects in apoptosis leading to the accumulation of dysfunctional cardiac cells [3] or conversely, excessive cell death [1].

It is possible that differences in the tissue-specific expression of mitochondrial proteins may explain part of the reason that more mitochondrial diseases do not exhibit cardiomyopathy [4]. For example, although the *HADH* gene is expressed in the heart, kidneys, liver, and muscle, it is most often expressed in the pancreatic islets [36], which is the site of primary disease manifestation [37].

It is also possible, especially for mitochondrial diseases with only a small number of reported patients, that this gene defect can cause cardiomyopathy but its presentation has simply not yet been reported. This could occur due to variable penetrance [38], lethal cardiomyopathy-associated variants [39], cardiomyopathy without a definitive genetic workup, the existence of cardiomyopathy in a patient with this gene defect that was not reported, or subclinical cardiomyopathy not detected due to a lack of screening [15,34]. For example, a patient with an ejection fraction of 45% will be asymptomatic, but this is not normal. In contrast, if neuronal signaling is affected even slightly, this is likely to cause noticeable consequences for behavior, development, and cognition, potentially explaining the much higher prevalence of neurological disease (93%) compared to cardiomyopathy (44%) reported for mitochondrial disease-causing genes in the Compendium and elsewhere [6,40].

Given that our analysis of the number of amino acids and number of physical interactors as surrogates of OXPHOS protein “importance” was not very revealing, we turned to animal models for a more mechanistic approach to our question. Animal models may offer important insights into why the proportion of mitochondrial diseases presenting with cardiomyopathies is relatively low. They can be particularly helpful in cases where the number of human patients reported with the disease is very small. The 23 genes for which we found both human and mouse cardiomyopathies may offer mechanistic insights into the causes underlying human cardiomyopathies and whether the cause is truly just an energy deficit in most cases. The 26 genes for which we found cardiomyopathies in mice but not in humans likely reflect that most mouse models are knockout models and are thus more severely affected than human patients, who may have mild cardiac dysfunction without a clinical phenotype. Moreover, human patients may be heterozygotes (AD inheritance) who would be haploinsufficient/hypomorphs. These mouse models may suggest potential human cardiomyopathies, particularly in those mitochondrial diseases that are especially rare. Therefore, these mouse models may provide opportunities to study human cardiac function in detail and to complete a thorough cardiac evaluation of humans with mutations in these genes.

Genetic background may also play an important role in whether or not a gene variant presents with a cardiomyopathy. For example, in mouse models, the presence or absence of *Nnt* can be a key factor in determining whether or not cardiomyopathy results [41,42]. Additionally, 12 of the 52 mouse models with cardiomyopathy that our study identified suggested that the presentation of cardiomyopathy may be dependent on the degree of stress. Given that many patients with mitochondrial diseases have skeletal muscle involvement, which results in decreased activity or immobility, they may not frequently encounter stress conditions that lead to the presentation of cardiac manifestations [3].

This study was an initial inquiry into the question of why more mitochondrial diseases do not result in cardiomyopathy. It used existing data and has several important limitations. First, the Mootha categories [25], and therefore this analysis, may not “capture” particular roles of a gene product since they classify each gene into one category based on its primary function. For example, *TAFAZZIN* can be thought of as playing a role in energy production, even though it is categorized under “Metabolism” and specifically, “Phospholipid Metabolism” [43]. Many other genes not categorized into OXPHOS may also lead to energy deficiencies if dysfunctional [5]. Indeed, our PubMed search found that 39/58 (67%) of the non-OXPHOS genes that caused cardiomyopathy were linked to deficits in energy production.

Second, the Compendium’s reported symptoms are based on the existing literature detailing manifestations of mitochondrial diseases rather than a systematic screening procedure. Therefore, it is possible that some mitochondrial genes that cause cardiomyopathy have not yet been reported in cases and are currently incorrectly understood not to cause cardiomyopathy. Third, the Compendium does not include information about how many patients were studied for each of the investigated genes or what proportion actually reported cardiomyopathy. Rather, it is a binary report of whether or not a particular gene associated with mitochondrial disease has had any known cases of cardiomyopathy, making the interpretation of the underlying data less nuanced. Fourth, this study utilized a statistical approach to look for differences between 241 mitochondrial genes causing versus not causing cardiomyopathy. Due to the small size of the subgroups compared (e.g., different gene categories, different gene subcategories), a statistical approach is inherently limited since it often depends on large sample sizes to ensure adequate power.

In the future, we believe that the rigorous characterization of mouse models and/or stem cell/iPSC-based approaches (iPSC-derived cardiomyocytes of human pathogenic variants) [44,45] will hold the key to determining how mitochondrial dysfunction affects the heart. Our study offers some support for the hypothesis that the mechanism underlying cardiomyopathy in patients with mitochondrial diseases is energy deficiency. However, many genes that affected energy production (i.e., OXPHOS defects) did not cause cardiomyopathy and many genes that did not directly affect energy production were found to indirectly cause issues with aerobic respiration in the literature. Moreover, many of the genes had mouse models that demonstrated cardiomyopathy although there were no known human cases of cardiomyopathy.

Eventually, each of these genes may need to be experimentally analyzed for the presence and degree of energy deficits. This should involve a systematic analysis of energy production, histology, and overall organ function. The role of factors such as protein function redundancy as well as residual function (hypomorphs) also warrants further exploration. Research on genetic background and how it affects the presentation of cardiomyopathy versus no cardiomyopathy may further explain differences seen among gene variants in the literature [41,42,46].

Continuing to identify and report the phenotypes of patients with mitochondrial diseases will also be key, especially for new or particularly rare diseases. To this end, increased screening for cardiac involvement in mitochondrial diseases could help identify more cases. While screening with an EKG and echocardiogram is currently recommended for all patients with primary mitochondrial disease [47,48], it may not always be completed [15,34]. Notably, in a review of 113 patients with mitochondrial diseases, patients with cardiomyopathy had an 18% survival rate at age 16 compared to patients without cardiomyopathy, who had a 95% survival rate at the same age [6]. Adults with mitochondrial disease are also at risk of poor outcomes. In a cohort of 260 patients aged ≥18 years, 30% had cardiac involvement at baseline and 10% had major adverse cardiac events over a follow-up period of seven years [49,50]. Increasing adherence to cardiac screening recommendations could help identify more cases to better understand the pathophysiology of mitochondrial diseases and help treat patients with cardiac diseases earlier.

## 5. Conclusions

A comprehensive analysis of genes associated with mitochondrial disease found that those involved in OXPHOS and fatty acid metabolism were linked to the highest proportion of cardiomyopathy and were significantly associated with cardiomyopathy. Furthermore, many of the non-OXPHOS genes linked to cardiomyopathy were found to be associated with defects in aerobic energy production in the literature. While this provides some support for the prevailing hypothesis that cardiomyopathy in mitochondrial diseases is caused by decreased energy production, it does not explain the large number of OXPHOS genes that were not associated with the development of cardiomyopathy. We explored reasons for why this might be, including differences in the tissue-specific expression of mitochondrial proteins, lethal cardiomyopathy-associated variants, and differences in genetic backgrounds. Finally, we suggest promising avenues for future research, including the increased development and systematic characterization of deficits in mouse models with mitochondrial diseases and the increased adherence to cardiac screening guidelines for children with mitochondrial diseases.

## Figures and Tables

**Table 1:** Characteristics of Mitochondrial Disease-Causing Genes by the Presence or Absence of Cardiomyopathy.

	Cardiomyopathy (*n* = 107)	No Cardiomyopathy (*n* = 134)	*p*-Test
Localization Category (%)			0.511
Gene Category (%)			<0.001
Oxidative Phosphorylation (OXPHOS)	**49 (45.8)**	**34 (25.4)**	**0.001**
OXPHOS Complex (%)			0.020
I	19 (38.8)	17 (50.0)	0.371
II	4 (8.2)	1 (2.9)	0.644
III	**0 (0.0)**	**5 (14.7)**	**0.010**
IV	8 (16.3)	6 (17.6)	1.000
V	4 (8.2)	0 (0.0)	0.141
Accessory	14 (28.6)	5 (14.7)	0.187
mtDNA Gene Expression	35 (32.7)	39 (29.1)	0.576
mtDNA Gene Expression Subcategory (%)			0.797
tRNA	10 (28.6)	12 (30.8)	1.000
tRNA Synthetase	6 (17.1)	11 (28.2)	0.284
Ribosome Component	4 (11.4)	3 (7.7)	0.701
Nucleotide Modification	5 (14.3)	4 (10.3)	0.727
Other	10 (28.6)	9 (23.1)	0.606
Metabolism	17 (15.9)	35 (26.1)	0.060
Metabolism Subcategory (%)			0.058
Intermediary Metabolism	6 (35.3)	13 (37.1)	1.000
Mitochondrial Fatty Acid Metabolism	**7 (41.2)**	**3 (8.6)**	**0.009**
Amino Acid Metabolism	1 (5.9)	8 (22.9)	0.241
Phospholipid Metabolism	2 (11.8)	3 (8.6)	1.000
Krebs/TCA Cycle	0 (0.0)	4 (11.4)	0.290
Nucleotide Metabolism	0 (0.0)	3 (8.6)	0.542
Other	1 (5.9)	1 (2.9)	1.000
Solute Carrier Family	**3 (2.8)**	**13 (9.7)**	**0.038**
Other	**1 (0.9)**	**11 (8.2)**	**0.014**
Protein Targeting to Mitochondria	2 (1.9)	2 (1.5)	1.000
Genome Origin (%)			0.188
Nuclear Gene	88 (82.2)	116 (86.6)	0.374
Nervous System Manifestations	101 (94.4)	123 (91.8)	0.462

Legend: **Red bold** indicates significant association (*p* < 0.05) with cardiomyopathy; **Blue bold** indicates significant association with NO cardiomyopathy. Localization (%) compares genes in the following locations: mitochondrial inner membrane, mitochondrion, mitochondrial matrix, mitochondrial ribosome in mitochondrial matrix, mitochondrial membrane, mitochondrial intermembrane space, mitochondrial outer membrane, and other. Genome origin (%) compares genes in the following locations: mitochondrion, nuclear chromosome 1, nuclear chromosome 2, etc.

## Data Availability

The data presented in this study are contained within the supplementary materials.

## Supplementary Materials

The following supporting information can be downloaded at: https://www.mdpi.com/article/10.3390/jcdd10040154/s1, Figure S1: Amino Acid and Physical Interactor Analysis of OXPHOS Genes, (a) Number of Amino Acids by the Presence of Cardiomyopathy, (b) Number of Physical Interactors by the Presence of Cardiomyopathy, (c) Relationship Between Protein Size and Number of Physical Interactors; Table S1: Mitochondrial Disease Genes Compendium with Additional Analysis; Table S2: Association of Non-OXPHOS Genes That Cause Cardiomyopathy And Known Defects in Aerobic Energy Production; Table S3: Mouse Models of Mitochondrial Genes; Table S4: International Mouse Phenotyping Consortium (IMPC) Data Compared to Mouse Model Data.
