# Supplementary material for: Why Don’t More Mitochondrial Diseases Exhibit Cardiomyopathy?
Source: J Cardiovasc Dev Dis. 2023 Apr 1;10(4):154. doi: 10.3390/jcdd10040154 (PMC10144188; doi:10.3390/jcdd10040154)
Supplement: Supplementary file 1 [file jcdd-10-00154-s001.zip › Figure S1.pdf]

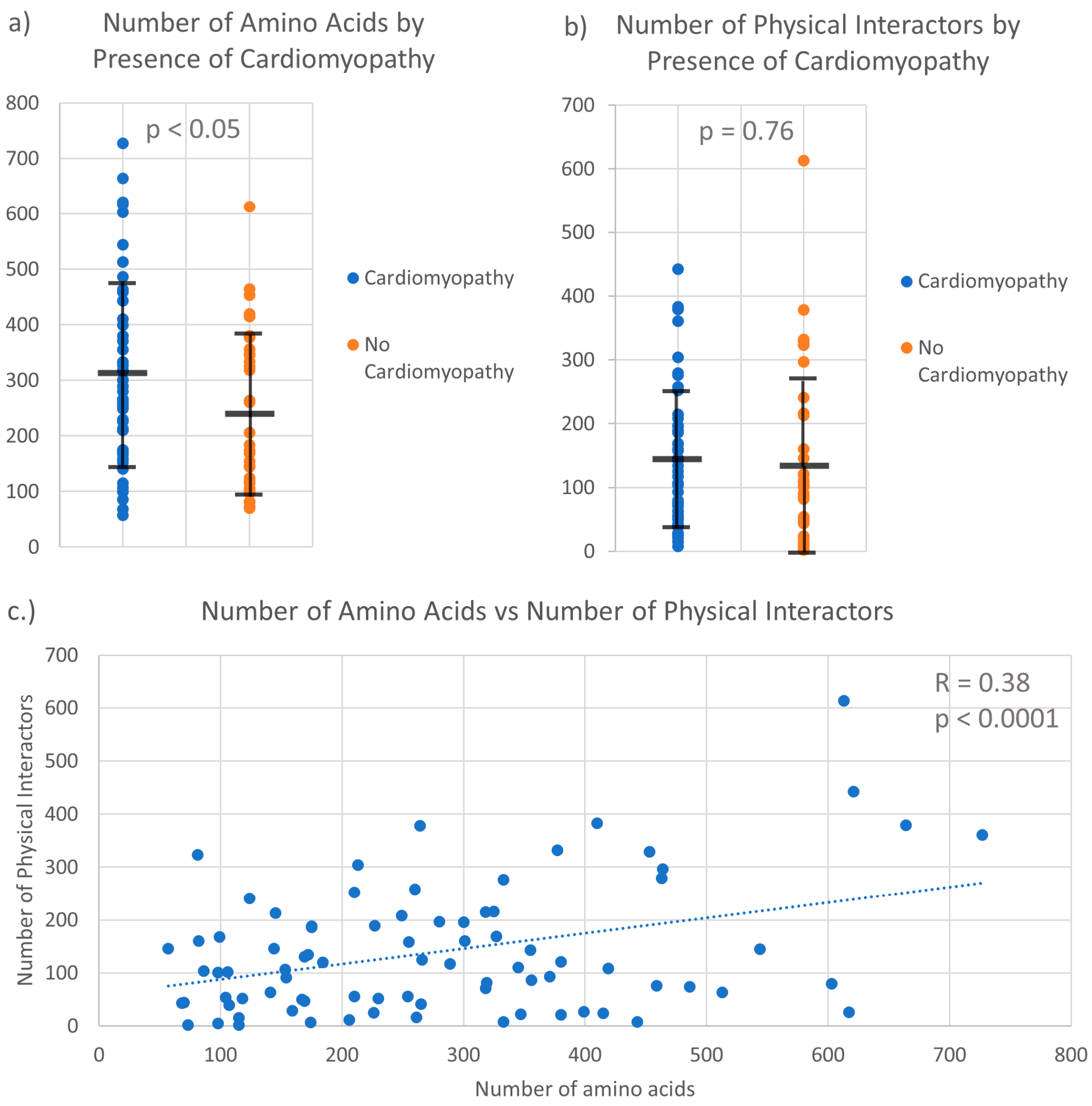


**Figure S1:** Amino Acid and Physical Interactor Analysis of OXPHOS Genes. (**a**) Number of Amino Acids by the Presence of Cardiomyopathy, (**b**) Number of Physical Interactors by the Presence of Cardiomyopathy, (**c**) Relationship Between Protein Size and Number of Physical Interactors.
